# Effects of positive end-expiratory pressure on lung ultrasound patterns and their correlation with intracranial pressure in mechanically ventilated brain injured patients

**DOI:** 10.1186/s13054-022-03903-7

**Published:** 2022-01-28

**Authors:** Chiara Robba, Lorenzo Ball, Denise Battaglini, Francesca Iannuzzi, Iole Brunetti, Pietro Fiaschi, Gianluigi Zona, Fabio Silvio Taccone, Antonio Messina, Silvia Mongodi, Paolo Pelosi

**Affiliations:** 1 Anesthesia and Intensive Care, San Martino Policlinico Hospital, IRCCS for Oncology and Neurosciences, Genoa, Italy; 2 Department of Surgical Sciences and Integrated Diagnostics (DISC), University of Genoa, Viale Benedetto XV 16, Genoa, Italy; 3 Department of Neurosurgery, San Martino Policlinico Hospital, IRCCS for Oncology and Neurosciences, Genoa, Italy; 4 Department of Intensive Care Medicine, Erasme Hospital, Université Libre de Bruxelles, Brussels, Belgium; 5 Humanitas Clinical and Research Center – IRCCS, Rozzano, MI Italy; 6 Dipartimento Di Medicina Intensiva, SC Anestesia e Rianimazione 1, Fondazione IRCCS Policlinico San Matteo, Pavia, Italy

**Keywords:** Intracranial pressure, Lung ultrasound, Positive end expiratory pressure, Brain injured patients, Mechanical ventilation

## Abstract

**Background:**

The effects of positive end-expiratory pressure (PEEP) on lung ultrasound (LUS) patterns, and their relationship with intracranial pressure (ICP) in brain injured patients have not been completely clarified. The primary aim of this study was to assess the effect of two levels of PEEP (5 and 15 cmH_2_O) on global (LUStot) and regional (anterior, lateral, and posterior areas) LUS scores and their correlation with changes of invasive ICP. Secondary aims included: the evaluation of the effect of PEEP on respiratory mechanics, arterial partial pressure of carbon dioxide (PaCO_2_) and hemodynamics; the correlation between changes in ICP and LUS as well as respiratory parameters; the identification of factors at baseline as potential predictors of ICP response to higher PEEP.

**Methods:**

Prospective, observational study including adult mechanically ventilated patients with acute brain injury requiring invasive ICP. Total and regional LUS scores, ICP, respiratory mechanics, and arterial blood gases values were analyzed at PEEP 5 and 15 cmH_2_O.

**Results:**

Thirty patients were included; 19 of them (63.3%) were male, with median age of 65 years [interquartile range (IQR) = 66.7–76.0]. PEEP from 5 to 15 cmH_2_O reduced LUS score in the posterior regions (LUSp, median value from 7 [5–8] to 4.5 [3.7–6], *p* = 0.002). Changes in ICP were significantly correlated with changes in LUStot (rho = 0.631, *p* = 0.0002), LUSp (rho = 0.663, *p* < 0.0001), respiratory system compliance (rho = − 0.599, *p* < 0.0001), mean arterial pressure (rho = − 0.833, *p* < 0.0001) and PaCO_2_ (rho = 0.819, *p* < 0.0001). Baseline LUStot score predicted the increase of ICP with PEEP.

**Conclusions:**

LUS-together with the evaluation of respiratory and clinical variables-can assist the clinicians in the bedside assessment and prediction of the effect of PEEP on ICP in patients with acute brain injury.

**Supplementary Information:**

The online version contains supplementary material available at 10.1186/s13054-022-03903-7.

## Background

The use of high positive end-expiratory pressure (PEEP) in brain injured patients has been challenged [1]. Concerns regarding the potential detrimental effects of PEEP on cerebral hemodynamics include different pathophysiological mechanisms: the risk for increased intrathoracic pressure with reduced jugular venous outflow, hemodynamic instability [1–4], alveolar overdistension with consequent increase of arterial partial pressure of carbon dioxide (PaCO_2_), resulting in reduced cerebral perfusion pressure (CPP) and higher intracranial pressure (ICP).

As increased ICP is associated with poor outcome [5], it is fundamental for neuro-intensive care unit (ICU) physicians to better understand and possibly predict the effect of PEEP on the brain. In fact, in the past, brain injured patients were often ventilated with no- or very low PEEP because of these concerns [6]. Literature is lacking on this topic, as demonstrated by a recent systematic review and consensus on mechanical ventilation in brain injured patients [1].

In a small physiological study using quantitative Computed Tomography (qCT) [7], we showed that PEEP could be safe in brain injured patients when promoting alveolar recruitment, without causing alveolar hyperdistention, decreased mean arterial pressure and cerebral blood flow. However, although qCT is the gold standard for the evaluation of the amount of collapsed lung tissue regaining inflation [8], it requires transfer of the patient to the CT facility, and carries the risk of radiation exposure. In addition, PEEP titration is performed often at patients’ bedside during ICU stay and during the day, thus making unfeasible the use of serial CTs to make the decision to increase PEEP. Lung Ultrasonography (LUS) is a safe, repeatable and easily available bedside technique [9]. LUS has been widely used in patients with acute respiratory distress syndrome (ARDS) and COVID-19 pneumonia with the aim to assess lung recruitment after PEEP application or recruitment maneuvers [9–12]. However, LUS has never been applied in brain injured patients to assess ultrasound patterns correlated with the response of ICP to PEEP.

Therefore, we conducted a prospective observational study on a population of mechanically ventilated brain injured patients to assess the effect of two levels of PEEP – 5 cmH_2_O and 15 cmH_2_O—on global and regional LUS scores and their correlation with the changes of ICP, with the aim to evaluate if LUS can provide similar information as qCT about the effects of PEEP on lung densities and ICP.

Secondary aims included the assessment of the effect of PEEP on respiratory mechanics, PaCO_2_ and hemodynamics; the correlation between changes in ICP and LUS; the identification of factors at baseline as potential predictors of ICP response at higher PEEP.

## Methods

This study is reported according to the “Strengthening the Reporting of Observational Studies in Epidemiology (STROBE)” statement guidelines for observational cohort studies (Additional file 1: ESM Table S1) [13] and was approved by the local ethics review board (Comitato Etico Regione Liguria, protocol n. CER Liguria: 23/2020). According to local regulations, written consent was obtained from patients’ next of kin, as all patients were unconscious at the time of inclusion.

### Inclusion and exclusion criteria

Patients were screened for inclusion from August 1st 2020 to September 1st 2021, and considered eligible if they were > 18 years old, required mechanical ventilation and were admitted to the ICU of San Martino Policlinico Hospital, IRCCS for Oncology and Neuroscience, Genoa, Italy, after acute brain injury (i.e. subarachnoid hemorrhage, SAH; traumatic brain injury, TBI; intracranial hemorrhage, ICH) requiring invasive ICP monitoring, and if underwent LUS evaluation based on clinical indications at two different levels of PEEP (5 and 15 cmH_2_0). Patients were excluded in case of absence of invasive ICP monitoring or informed consent, or if they did not receive LUS or PEEP test from 5 to 15 cmH_2_0.

### Data collection

Patients’ data were extrapolated from electronic clinical records and included admission demographics [i.e., age, sex, body mass index (BMI)], pre-injury comorbidities (i.e., respiratory, cardiological, kidney, metabolic diseases), type of acute brain injury (TBI, SAH, ICH), Glasgow Coma Scale (GCS) at ICU admission, type of ICP monitoring inserted (intraparenchymal or external ventricular drain), the occurrence of pulmonary and extrapulmonary ICU complications (i.e., sepsis, ventilator-associated pneumonia, acute kidney injury, other organs failure), and patients’ outcome, such as ICU length of stay, mortality and neurological status (as for Glasgow Outcome Score, GOS) at ICU discharge.

### Patients’ clinical management

In the ICU, patients were sedated with propofol and/or midazolam and fentanyl, were intubated and mechanically ventilated in pressure or volume-controlled ventilation. Tidal volume was targeted to 6–8 mL per kg of predicted body weight (PBW); however, higher values of tidal volume were tolerated, if driving pressure was maintained below 15 cmH_2_O.

### PEEP test and measurement of LUS and respiratory mechanics

The decision to perform a PEEP test was based on the clinician’s evaluation if optimization of mechanical ventilation was required, according to local protocols. PEEP test was performed in volume-controlled ventilation in all patients, under strict monitoring of ICP, without neuromuscular blockade. So far, no specific indications are available on the optimal levels of PEEP to be applied in brain injured patients [1]. A recent consensus [1] recommended to use the same level of PEEP applied in the non-brain injured population, and thus, in our center, in patients with deteriorating respiratory function a PEEP test is performed by slowly increasing PEEP (about 2 cmH_2_O every minute), from 5 to 15 cmH_2_O, and evaluating step by step the changes in respiratory mechanics and cerebral hemodynamics to set the optimal values of PEEP. Previous evidence and our clinical experience suggest that these values of PEEP are safe in brain injured patients [3, 7, 14].

Data were obtained at PEEP 15 cmH_2_O after allowing 5 min for stabilization. We used this relatively short time for high PEEP exposure before repetition of the cerebral and respiratory measurements, as previous studies showed that the majority of changes in volume and recruitment occurs in this timeframe and that most respiratory units recruit below 30 cmH_2_O [15, 16]. The chosen levels of PEEP (5 and 15 cmH_2_O) represent the standard levels of PEEP used to estimate response to PEEP in ARDS patients [17].

Data collected at PEEP 5 and PEEP 15 cmH_2_O included regional and global LUS score, as well as neuromonitoring parameters (ICP, optic nerve sheath diameter (ONSD), systolic (FVs), mean (FVm), and diastolic Flow Velocities (FVd); respiratory mechanics and arterial blood gases parameters including arterial partial pressure of oxygen (PaO_2_)/inspired fraction of oxygen (FiO_2_) ratio, tidal volume (*V*_T_), plateau pressure (*P*plat), Crs, respiratory rate (RR), arterial saturation of oxygen (SaO_2_), arterial pH (pHa) and PaCO_2_.

### Lung ultrasound

LUS was performed using a linear (12 MHz) or phased-array probe (2.5 MHz) for the visualization of pleural line or tissue-like pattern, respectively [12, 18, 19]. For each hemithorax, six regions were explored (upper and lower parts of anterior, lateral, and posterior chest wall). LUS videos were analyzed by two expert physicians (S.M., C.R.), and each clip was analyzed by two operators, whereas discordant clips were evaluated by a third. Analyzers were blinded to patients’ demographics and clinical status, PEEP level, and CT findings. Four ultrasound aeration patterns were defined with different scores: A line alone or less than three B lines (0 point); B lines with pleural involvement < 50% (1 point); B lines with pleural involvement > 50% (2 points), and lung consolidation (3 points) [20]. The worst ultrasound abnormality detected was considered as characterizing the region examined. The LUS score of each region (regional LUS score) corresponded to the average score of all pertaining intercostal spaces and ranged from 0 to 3. LUS score was then calculated globally (as the sum of the 12 regions score, ranging from 0 to 36), and regionally (LUSp, posterior, LUSa, anterior and LUSl, lateral regions). Lung ultrasound variations (ΔLUS) were computed as LUS score at PEEP 15—LUS score at PEEP 5 cmH_2_O; therefore, a reduction in lung ultrasound and negative values of ΔLUS will correspond to an increase in lung aeration at higher PEEP levels. On the contrary, an increase in LUS score and positive values of ΔLUS will correspond to a reduction in lung aeration at PEEP 15 cmH_2_O. The presence of air bronchogram and its characteristics (static/dynamic/punctiform/arborescent) was also reported.

### Neuromonitoring

Invasive ICP monitoring was inserted according to our local policies and clinical practice, and following the latest Brain Trauma Foundation Guidelines [21]. Ultrasound measurements were performed after PEEP augmentation, contemporarily to the LUS evaluation. A selected group of operators (FI, LB, DB) with extensive experience in brain ultrasonography performed the optic nerve sheath diameter (ONSD) and transcranial Doppler (TCD) measurements. Ultrasound examination of the ONSD was performed using a 7.5 MHz linear ultrasound probe (Philips SparQ®), as previously described [22], with patients in supine position. Four measurements were obtained for each patient, in the axial and sagittal planes of both eyes, and the widest diameter visible 3 mm behind the retina was considered. The final ONSD value was calculated as the mean of the four values [22–24]. TCD was performed bilaterally through the temporal window, on the middle cerebral artery (MCA), using a phased array 2-MHz transducer (Philips SparQ®, Amsterdam, Netherlands) [25, 26]. Non-invasive ICP estimation based on TCD (ICP_TCD_) was calculated using a previously validated formula [27].

### Statistical analysis

We were not able to perform an a priori sample size calculation as no studies on the effect of PEEP on LUS characteristics and ICP in brain injured patients are available. However, our sample size was higher compared to previous physiologic studies regarding PEEP augmentation in ARDS or in brain injured patients [28], and of a recent study using qCT for the assessment of recruitment in brain injured patients [7]. Data are reported as median (interquartile range, IQR), if not otherwise specified. Variables obtained at two levels of PEEP were compared using the Wilcoxon signed-rank test. Changes of variables from PEEP 5 to PEEP 15 were calculated as Δ (value at PEEP 15 cmH_2_O—value at PEEP 5 cmH_2_O). Correlations were sought using the Spearman’s rho. We further modeled ΔICP using linear regression as function of clinically sound covariates, adopting a variance inflation factor threshold of 5 as acceptable limit for multi-collinearity. All statistical analyses were performed in SPSS Statistics, Version 25.0 (IBM Corp., Armonk, NY, USA). Significance was assumed at two-tailed *p* < 0.05.

## Results

### Characteristics of the population

Over the described period, 42 patients were considered for inclusion. Among these, 11 were not monitored with invasive ICP, and one patient had no consent signed and was not included. A total of 30 patients were included in the final analysis. Four of them were also enrolled in our previous study [7]. Among these, 19 (63.3%) were males, with a median age of 65 years [51–73] (Table 1). Eighteen patients (60%) were admitted for TBI, 9 (30%) for SAH, and 3 (10%) for ICH. The median GCS score was 8 [3–12]. Five patients (16.6%) died in the ICU, and median GOS at ICU discharge was 4 [3, 4].

**Table 1:** Characteristics of the patients included in the study

Characteristics of patients	All patients (*n* = 30)
*Demographics*	
Gender, male [*n*, (%)]	19 (63.3%)
Age [years], median [IQR]	65 [51–73]
BMI [kg/m^2^], median [IQR]	26 [24–29]
PBW [kg], median [IQR]	70 [67–76]
*Comorbidities*	
Respiratory disease [*n*, (%)]	8 (26.6)
Cardiovascular disease [*n*, (%)]	4 (13.3)
Cancer [*n*, (%)]	1 (3.3)
Neurologic disorders [*n*, (%)]	1 (3.3)
Moderate/severe liver disease [*n*, (%)]	1 (3.3)
Chronic kidney injury [*n*, (%)]	1 (3.3)
Hypertension [*n*, (%)]	12 (40)
Diabetes mellitus [*n*, (%)]	3 (10)
*Reason for ICU admission [n, (%)]:*	
TBI	18 (60)
SAH	9 (30)
ICH	3 (10)
*GCS score at ICU admission, median [IQR]*	8 [3–12]
*Type of ICP monitor*	
Bold	17 (56.6)
EVD	13 (43.3)
Need for vasopressors [*n*, (%)]	13 (43.3)
*ICU complications*	
Acute Distress Respiratory syndrome [*n*, (%)]	1 (3.3)
Ventilator- associated pneumonia [*n*, (%)]	11 (36.6)
Cardiovascular [*n*, (%)]	3 (10)
Acute kidney injury [*n*, (%)]	1 (3.3)
Sepsis [*n*, (%)]	6 (20)
Vasospasm [*n*, (%)]	3 (10)
*ICU discharge characteristics*	
Mortality [*n*, (%)]	5 (16.6)
GOS, median [IQR]	4 [3, 4]
ICU length of stay, median [IQR]	18 [10–26]
ICU duration of mechanical ventilation, days [IQR]	10 [7–14]
Days of vasopressors administration [IQR]	6 [3–11]

*IQR* interquartile range, *n *number, *BMI* body mass index, *PBW* predicted body weight, *ICU* intensive care unit, *TBI* traumatic brain injury, *SAH* subarachnoid hemorrhage, *ICH* intracranial hemorrhage, *GCS* Glasgow Coma Scale, *ICP* intracranial pressure, *EVD* external ventricular drain, *GOS* Glasgow Outcome Score

### Effect of PEEP increase on lung ultrasound findings

**Fig. 1:**
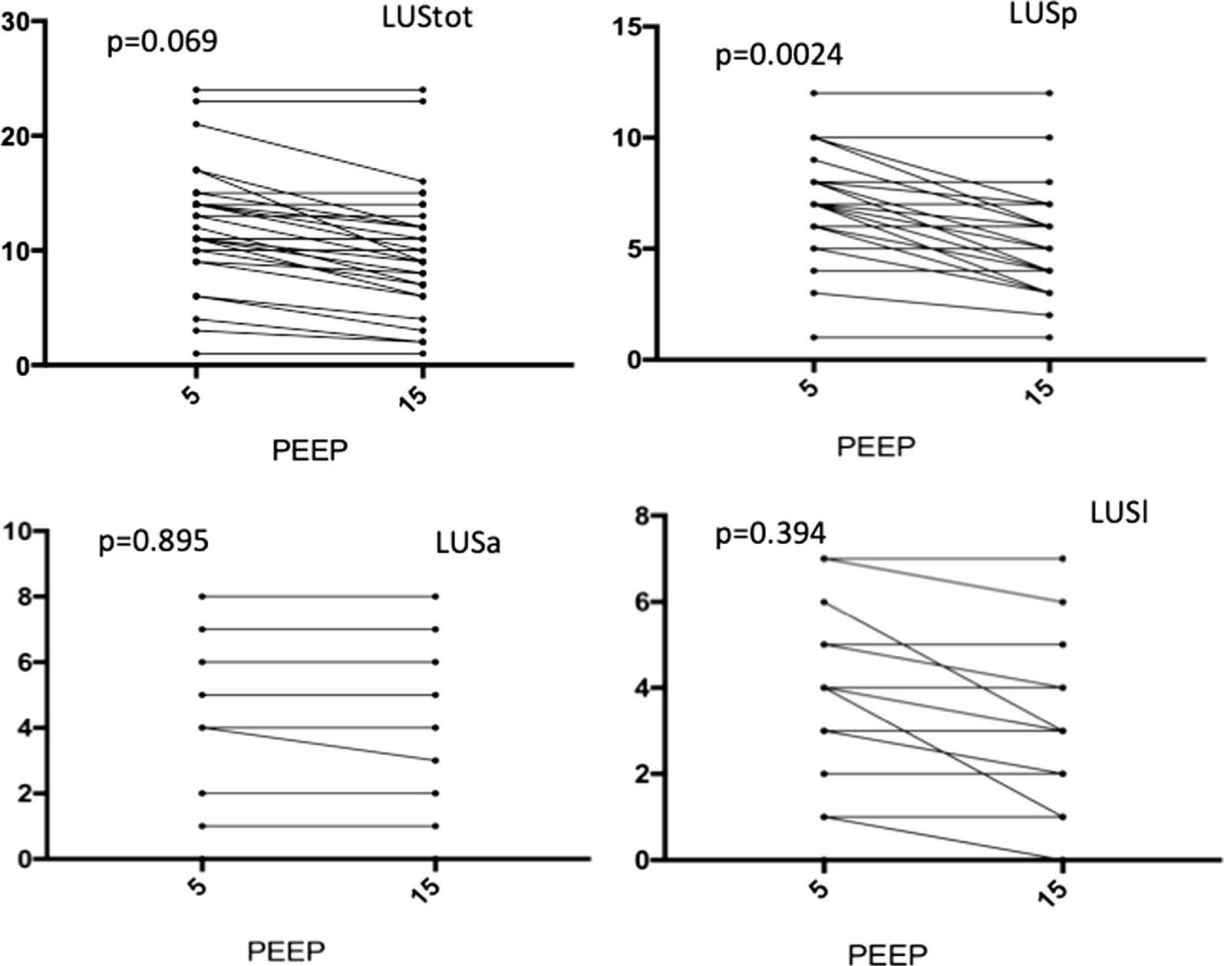
Total Lung ultrasound (LUS tot), and regional LUS score in the posterior, lateral and anterior regions of the lung (LUSp, LUSl, LUSa) at PEEP of 5 and 15 cmH_2_O. Black dots and lines represent individual patient data. PEEP: positive end-expiratory pressure

**Fig. 2:**
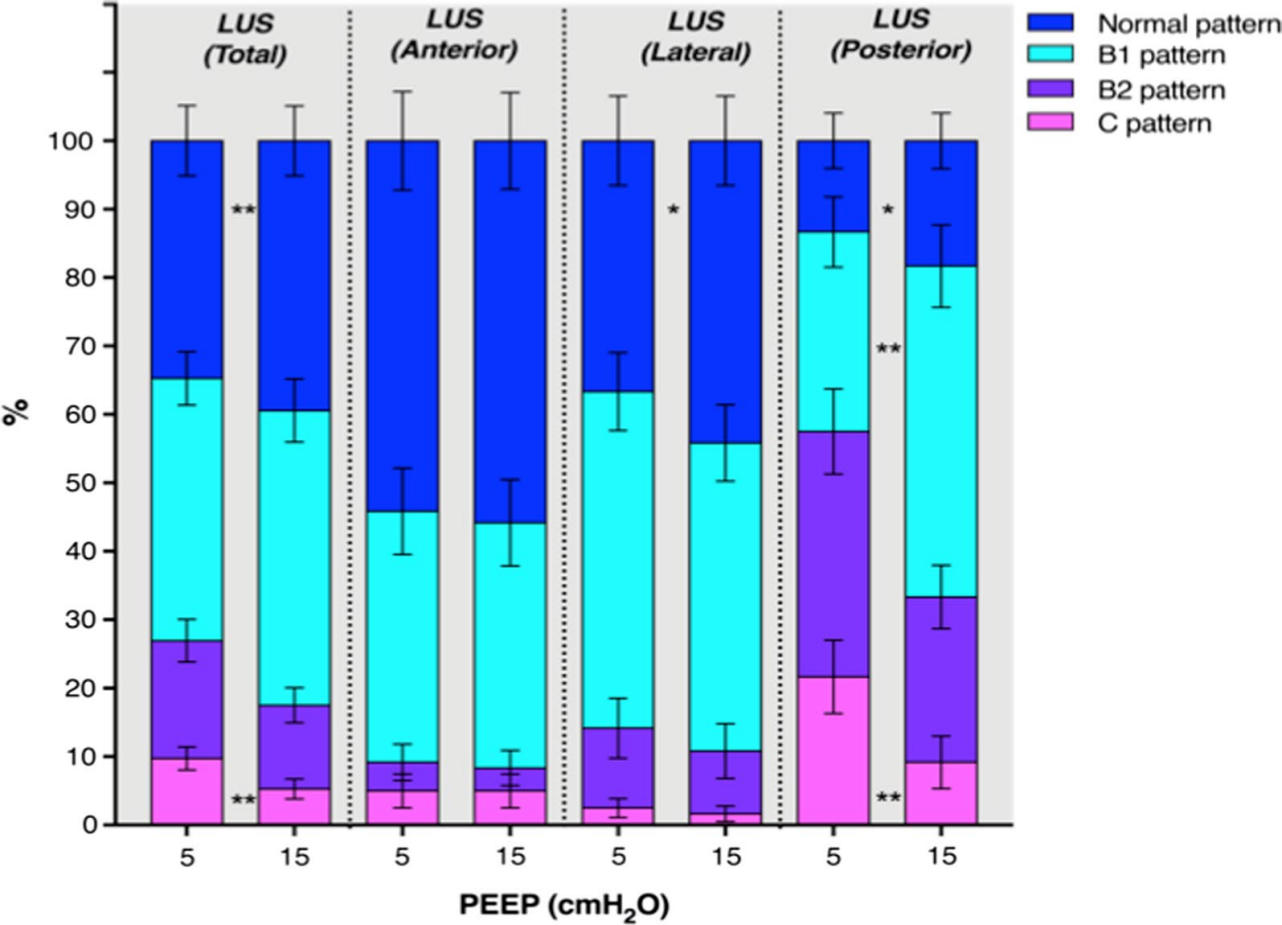
Quantitative LUS analysis in the whole lung and in the posterior, lateral and anterior regions of interest, considering the different LUS patterns (score 0–3) at PEEP 5 and 15 cmH_2_0. *p < 0.05; **p < 0.0001

After PEEP augmentation, total LUS score did not change significantly (from 12.5 [9.7–15] to 9.5 [6.7–13.2], *p* = 0.069) (Table 2, Fig. 1). Considering regional LUS score, LUSp was significantly lower after PEEP augmentation (7 [5–8] vs. 4.5 [3.7–6], *p* = 0.002) while no differences were found in LUSl and LUSa (3 [1–5] vs. 3 [1–4], *p* = 0.394 and 2 [0–4] vs. 2 [0–4], *p* = 0.895, respectively). Considering LUStot, the percentages of score 0 pattern were increased (*p* = 0.005), while in score 3 pattern were reduced (*p* = 0.007) after PEEP augmentation (Fig. 2). In the anterior areas, no differences were found in the LUS patterns increasing PEEP from 5 to 15 cmH_2_O. In the lateral areas, only the percentage of score 0 was increased after PEEP augmentation (*p* = 0.011). In the posterior areas, scores 0 and 1 were increased after PEEP augmentation (*p* < 0.014 and *p* < 0.002, respectively) while score 3 was reduced (*p* = 0.006). Dynamic punctiform air bronchogram was observed in ten patients. Punctiform static air bronchogram and arborescent static bronchogram were detected in two and six cases, respectively. Dynamic punctiform air bronchogram was present only in patients who did not experience increased ICP after PEEP augmentation.

**Table 2:** Total and regional Lung Ultrasound scores at PEEP 5 and 15 cmH_2_0

	PEEP = 5 (*N *= 30)	PEEP 15 (*N* = 30)	*p*
Total LUS score	12.5 [9.7–15]	9.5 [6.7–13.2]	0.069
Right lung			
R1	0 [0–1]	0 [0–1]	> 0.999
R2	0 [0–1]	0 [0–1]	0.703
R3	1 [0–1]	0.5 [0–1]	0.673
R4	1 [0–1]	1 [0–1]	0.532
R5	1 [0–2]	1 [0–1]	0.216
R6	2 [2, 3]	1 [1, 2]	0.0018
Left lung			
L1	0 [0–1]	0 [0–1]	> 0.999
L2	0.5 [0–1]	0.5 [0–1]	> 0.999
L3	1 [0–1]	0.5 [0–1]	0.752
L4	1 [0.75–1]	1 [0–1]	0.802
L5	1 [1, 2]	1 [0.7–1.2]	0.119
L6	2 [1–3]	1 [1–2.2]	0.089
Posterior lung regions (R5, R6, L5, L6)	7 [5–8]	4.5 [3.7–6]	0.002
Lateral lung regions (R3, R4, L3, L4)	3 [1–5]	3 [1–4]	0.394
Anterior lung regions (R1, R2, L1, L2)	2 [0–4]	2 [0–4]	0.895

Data are presented as median and Interquartile Range [IQR]

*PEEP* positive end expiratory pressure, *L* left, *LUS* lung ultrasound, *R* right. Variables obtained at two levels of PEEP were compared using the Wilcoxon signed-rank test

### Effect of PEEP on respiratory function, ICP and CPP

After PEEP augmentation, Pplat increased (from 21 [19–23] to 29 [28–31] cmH_2_0, *p* < 0.0001), and PaO_2_/FiO_2_ improved (from 182 [172–199] to 206 [196–242], *p* = 0.049), whereas no effect on PaCO_2_ and Crs was observed (from 38 [36–40] to 39 [37–41] mmHg, *p* = 0.341, from 31.3 [28–36] to 35 [32–40] ml/cmH_2_O, *p* = 0.200, respectively) (Additional file 1: Figure ESM 1 and Table 3). Invasive ICP and CPP did not significantly change (from 13 [5–16] to 16 [8–18] mmHg, p = 0.280; from 72 [62–79] to 66 [63–72] mmHg, p = 0.364, respectively).

**Table 3:** Ventilator settings, arterial blood gases values, neuromonitoring data and hemodynamics in our cohort at PEEP = 5 and 15 cmH_2_0

Parameter	PEEP = 5 (*N* = 30)	PEEP =15 (*N* = 30)	*p* value
Ventilator settings/arterial blood gases			
Plateau pressure, median [IQR], cmH_2_O	21 [19–23]	29 [28–31]	< 0.0001
Respiratory system compliance, median [IQR], ml/cmH_2_O	31.3 [28–36]	35 [32–40]	0.2
pHa, median [IQR]	7.35 [7.35–7.37]	7.36 [7.35–7.4]	0.914
PaO_2,_ median [IQR], mmHg	91 [86–100]	103 [98–121]	0.049
SaO_2,_ median [IQR], %	94 [93–96]	96 [95–97]	0.627
PaCO_2,_ median [IQR], mmHg	38 [36–40]	39 [37–41]	0.341
PaO_2_/FiO_2_, median [IQR]	182 [172–199]	206 [196–242]	0.049
Neuromonitoring			
ICP, median [IQR], mmHg	13 [5–16]	16 [8–18]	0.280
CPP,median [IQR], mmHg	72 [62–79]	66 [63–72]	0.364
FVs, median [IQR], cm/sec	112 [96–119]	104 [87–110]	0.243
FVd, median [IQR], cm/sec	30 [19–51]	24 [22–39]	0.176
FVm, median [IQR], cm/sec	59 [51–69]	53 [48–64]	0.212
ONSD median [IQR], mm	4.2 [3.9–4.8]	4.8 [4.3–5.2]	0.783
ICP_TCD,_ median [IQR], mmHg	15 [10–19]	18 [16–22]	0.084
PI, median [IQR]	0.8 [0.6–1.1]	1.2 [0.9–1.3]	0.091
Hemodynamics			
Mean arterial pressure, median [IQR], mmHg	86 [78–93]	83 [76–95]	0.885

*CPP* cerebral perfusion pressure, *FVs, FVd, FVm* systolic, diastolic, mean flow velocity, *ICP* intracranial pressure, *ICP*_*TCD*_ intracranial pressure measured with transcranial Doppler (TCD), *IQR* interquartile range, *ONSD* optic nerve sheath diameter, *PaCO*_*2*_ partial pressure of carbon dioxide, *PaO*_*2*_ partial pressure of oxygen, *SaO*_*2*_ arterial oxygen saturation, PaO_2_/inspired fraction of oxygen, FiO_2_, *PI* pulsatility index. Data are presented as median and Interquartile Range [IQR]. Variables obtained at two levels of PEEP were compared using the Wilcoxon signed-rank test

### Correlations between the changes of ICP with LUS patterns and respiratory mechanics

**Fig. 3:**
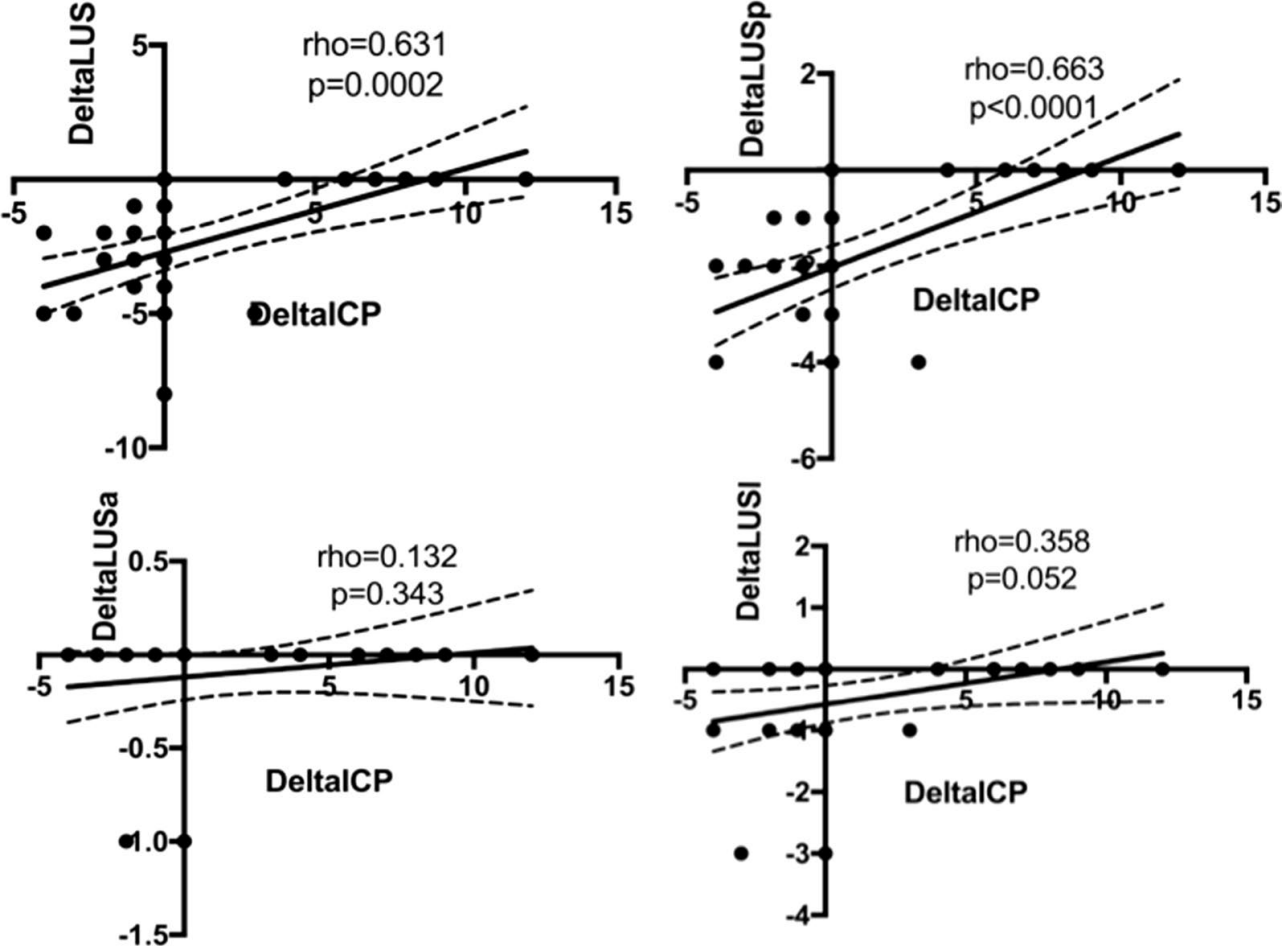
Scatterplots showing the linear association and correlation between Δ total Lung ultrasound (LUS) (left upper panel), ΔLUS posterior (right upper panel), ΔLUS anterior (left lower panel), ΔLUS lateral (right lower panel) versus Δ intracranial pressure (ICP) at different study timepoints. Dotted lines represent the 95% confidence intervals for the linear regression

ΔICP was correlated with ΔLUStot (rho = 0.631, *p* = 0.0002) and ΔLUSp (rho = 0.663, *p* < 0.0001), but not with ΔLUSa and ΔLUSl (rho = 0.179, *p* = 0.343 and rho = 0.358, *p* = 0.052, respectively) (Fig. 3). ΔICP was also significantly correlated with ΔCrs (rho = − 0.599, *p* < 0.0001), Δ*P*plat (rho = 0.771, *p* < 0.0001) ΔMAP (rho = − 0.833, *p* < 0.0001), and ΔPaCO_2_ (rho = 0.819, *p* < 0.0001) (Additional file 1: Figure ESM 2, 3). ΔLUStot and ΔLUSd were inversely correlated with ΔCrs (rho = v0.6830; *p* < 0.0001 and rho = − 0.7557; *p* < 0.0001, respectively), whereas ΔLUSl and ΔLUSa were not (rho = − 0.2966, *p* = 0.1115; rho = − 0.2539, *p* = 0.1758, respectively). Finally, ΔICP was correlated with ΔONSD (rho = 0.411, *p* = 0.024), but not with ΔICP_TCD_.

At linear regression analysis, basal LUStot was correlated with the changes in ICP after PEEP increase (regression coefficient B = − 2.378, 95% confidence interval CI from − 2.137 to − 0.413 to, *p* = 0.044). We did not observe any significant correlation between the basal values (at PEEP = 5 cmH_2_O) of ICP, ONSD, ICP_TCD_, Crs, MAP, PaCO_2,_ and the changes of ICP at PEEP 15 cmH_2_O.

## Discussion

In a cohort of mechanically ventilated brain injured patients undergoing a PEEP test from 5 to 15 cmH_2_O, we found that: (1) the improvement in LUS score occurs mainly in the posterior areas and is correlated with a decrease or no changes of ICP; (2) decreased respiratory system compliance and mean arterial pressure as well as increased PaCO_2_ are correlated with greater ICP augmentation; (3) basal LUStot can predict ICP increase after PEEP application. In particular, the higher is the basal LUStot, the smaller is the change of ICP after PEEP application.

This is the first study describing the use of LUS in brain injured patients for the definition of lung echographic patterns, and which assesses the effect of PEEP increase on ICP variation.

Little is known about the optimal levels of PEEP to be applied in mechanically ventilated brain injured patients [1]. The use of high PEEP has been previously discouraged in this population [6], as it can potentially increase intrathoracic pressure, reduce jugular veins outflow, and cause hemodynamic instability with consequent detrimental effects on CPP and cerebral blood flow. As impairment of CPP, ICP and cerebral blood flow is associated with poor outcome [5], traditionally, brain injured patients have been often ventilated with zero-positive end expiratory pressure (ZEEP) [6]; more recently, clinical practice has significantly changed, as the application of moderate PEEP has demonstrated to reduce the risk of atelectrauma and therefore ventilator induced lung injury and pulmonary complications [6]. Small physiological studies have also demonstrated that PEEP can be safe even in the neurocritical care population, as long as respiratory system compliance is not impaired (without increases in PaCO_2_), and hemodynamic stability is maintained [7, 28, 29].

In a recent study, we demonstrated that alveolar recruitment evaluated through qCT was correlated with increased ICP [7]. Although preliminary, these results suggest that PEEP can be safe in acute brain injured patients if it promotes alveolar recruitment, and if it does not affects Crs, or PaCO_2_. This is also in line with the current recommendations in this population of patients, which suggest to apply the same level of PEEP as for the general ICU population [1]. Although quantitative CT is the gold standard for the evaluation of alveolar recruitment [8], the need for transfer to radiology unit and the exposure to radiations preclude its routine use in all ICU patients, where PEEP titration is often performed, and it is therefore reserved to few specific cases. In contrast, LUS is a bedside, non-invasive, easily available technique which has shown to be able to assess lung morphology with precision in other populations [30, 31]. Compared to qCT, the currently used LUS scores have important limitations in the clinical settings, as they did not demonstrate a strong correlation with alveolar recruitment and they are not able to assess and evaluate areas of hyperinflation [12]. However, when compared to other techniques as pressure–volume curve, or recruitment to inflation ratio in populations of acute respiratory distress syndrome and in COVID-19, a significant correlation between LUS score variations and recruitment has been reported [32, 33].

Our study suggests that LUS can be used as surrogate of qCT to evaluate lung recruitment after PEEP increase and its effect on ICP, and that when PEEP increases lung volume by alveolar recruitment, the increase in plateau pressure is limited. This phenomenon is particularly relevant in the posterior areas of the lung, where we observed the highest percentage of pathologic LUS pattern and in particular lung densities (score 3), potentially able to respond to recruitment. A possible explanation in these cases is that, independently from individual variations in the chest wall compliance, the transmission of airway pressure to the thoracic compartment is reduced, thus minimally affecting increases in ICP. On the other side, if PEEP overinflates aerated lung without alveolar recruitment, Crs decreases, yielding greater increase in Pplat. In this case, the transmission of airway pressure to the thoracic compartment is increased, thus promoting an increase in ICP.

LUS scores in the anterior and the lateral areas of the lung did not importantly change after PEEP application and were not correlated with ICP changes.

This is a new finding, when compared to the available literature on the use of lung ultrasound for PEEP titration, which reports in fact the interest in the loss of aeration of mainly the anterior fields to identify PEEP responders [31]. This can be explained by the fact that previous studies focused on patients with ARDS, where posterior fields are almost constantly affected by a severe or complete loss of aeration. In our population of brain injured patients, posterior fields were on the contrary the most informative to predict the positive response to PEEP, probably because the most frequent causes of loss of aeration are atelectasis and superinfection, especially located in posterior areas in supine position, while anterior fields showed almost constantly a normal aeration.

Moreover, in our population, we observed median lower LUS scores than what reported by previous literature on patients with acute respiratory failure [31]; this suggests that previously proposed cut-off values are likely to be not appropriate in different ICU populations.

In the present study, none of the respiratory and neuromonitoring derived parameters evaluated at baseline PEEP was predictive for ICP increase with PEEP. However, a higher LUS score at baseline was associated with a reduced risk of changes in ICP, thus suggesting that in patients with greater severity of lung injury and pulmonary morphology (with higher incidence of consolidations), PEEP can cause recruitment without causing important effects on cerebral hemodynamics. A signal on the consolidations features is also present, with higher potential for PEEP responsiveness in consolidations with dynamic punctiform air-bronchogram, corresponding to patent airways with airflow during inspiration.

We also found a significant correlation between changes of ONSD and ICP, thus confirming our previous findings [7], and suggesting that ONSD can be used when ICP is not available or not indicated to evaluate ICP fluctuation related to manipulation of intrathoracic pressure [34, 35].

In summary, in this study we demonstrated that LUS can be used as a surrogate of qCT to evaluate the effect of PEEP on ICP and can be used at patient’s bedside to predict the effect of PEEP on ICP. Despite our sample size is limited, this represents a great novelty as LUS can become an important clinical tool for neuro-ICU physicians. By performing serial bedside LUS, they would be able to safely titrate PEEP and assess/predict the effect of PEEP on intracranial pressure, minimizing the risk of intracranial hypertension and secondary brain damage. LUS is a radiation free, non-invasive, safe and easily available tool; however, it can present some limitations compared to qCT (for instance, it cannot identify lung overinflation, it is difficult to use in obese patients, it is an operator-dependent technique) [36, 37].

### Limitations

This study has several limitations that need to be mentioned. First, in our ICU, the PEEP test is a routine procedure that is normally performed but only in selected patients with acute brain injured patients, and when the PaO_2_/FiO_2_ is < 300 at PEEP of 5 cmH_2_O.  In addition, we included patients with different types of brain damage, thus resulting in a heterogeneous population for both cerebral and respiratory characteristics. Second, although we standardized mechanical ventilator settings, respiratory mechanics evaluation and arterial blood gases measurement during PEEP test, we cannot exclude that different ventilator setting may have led to different results [1, 38]. Third, this study would have benefit from more details and information on cardiac function and hemodynamic monitoring, which would have helped in the understanding of the interplay between lung, heart, and brain after PEEP application. However, this was an observational study, and it is not our routine practice to perform echocardiography or carotid flow assessment during PEEP test. Fourth, although the sample size is greater compared to other similar physiological studies on the effect of PEEP on lung recruitment, the small number of patients in our cohort cannot be used to draw conclusions or to assume consideration and/or strong statements on this topic. Larger studies are needed to confirm our results [28, 39, 40].

## Conclusions

LUS, together with the evaluation of respiratory variables, can assist the clinicians in the bedside assessment and prediction of the effect of PEEP on ICP in mechanically ventilated patients with acute brain injury.

Further larger studies are warranted to assess the role of LUS for the titration of PEEP in this cohort of patients, and to create a statistical model with selected independent variables aimed to improve the accuracy of the prediction of ICP response to PEEP.

## Supplementary Information

**Additional file 1.** STROBE, additional information on data analysis, methods, and additional results.

## Data Availability

The datasets used and/or analyzed during the current study are available from the corresponding author on reasonable request.

## Abbreviations

ARDS: Acute respiratory distress syndrome
BMI: Body mass index
CPP: Cerebral perfusion pressure
Crs: Respiratory system compliance
ESM: Electronic Supplementary materials
FiO2: Inspired fraction of oxygen
FVd: Diastolic Flow Velocity
FVm: Mean Flow Velocity
FVs: Systolic Flow Velocity
GCS: Glasgow Coma Scale
GOS: Glasgow Outcome Score
ICH: Intracranial hemorrhage
ICP: Intracranial pressure
ICP_TCD_: Intracranial pressure measured through Transcranial Doppler
ICU: Intensive care unit
IQR: Interquartile range
LUS: Lung ultrasonography
LUSa: LUS anterior
LUSl: LUS lateral
LUSp: LUS posterior
MCA: Middle cerebral artery
ONSD: Optic nerve sheath diameter
PaCO_2_: Arterial partial pressure of carbon dioxide
PBW: Predicted body weight
PEEP: Positive end-expiratory pressure
pHa: Arterial pH
Pplat: Plateau pressure
qCT: Quantitative computed tomography
RR: Respiratory rate
SAH: Subarachnoid hemorrhage
SaO_2_: Arterial saturation of oxygen
STROBE: Strengthening the reporting of observational studies in epidemiology
TBI: Traumatic brain injury
TCD: Transcranial doppler
VT: Tidal volume
ΔLUS: Lung ultrasound variations
