# Supplementary material for: Effects of positive end-expiratory pressure on lung ultrasound patterns and their correlation with intracranial pressure in mechanically ventilated brain injured patients
Source: Crit Care. 2022 Jan 28;26:31. doi: 10.1186/s13054-022-03903-7 (PMC8796179; doi:10.1186/s13054-022-03903-7)
Supplement: Supplementary file 1 — Additional file 1. STROBE, additional information on data analysis, methods, and additional results. [file 13054_2022_3903_MOESM1_ESM.docx]

Additional file 1

Effects of positive end-expiratory pressure on lung ultrasound patterns and their correlation with intracranial pressure in mechanically ventilated brain injured patients

Chiara Robba^1,2^, Lorenzo Ball^1,2^, Denise Battaglini^1^, Francesca Iannuzzi^1^, Iole Brunetti^1^, Pietro Fiaschi^3^, Gianluigi Zona^3^, Fabio Silvio Taccone^4^,Antonio Messina^5^,Silvia Mongodi^6^ and Paolo Pelosi^1,2^

^1^Anesthesia and Intensive Care, San Martino Policlinico Hospital, IRCCS for Oncology and Neurosciences, Genoa, Italy

^2^Department of Surgical Sciences and Integrated Diagnostics (DISC), University of Genoa, Genoa, Italy

^3^ Department of Neurosurgery, San Martino Policlinico Hospital, IRCCS for Oncology and Neurosciences, Genoa, Italy

^4^Department of Intensive Care Medicine, Erasme Hospital, Université Libre de Bruxelles, Brussels, Belgium

^5^ Humanitas Clinical and Research Center – IRCCS, Rozzano, MI, Italy

^6^ Dipartimento di Medicina Intensiva, SC Anestesia e Rianimazione 1, Fondazione IRCCS Policlinico San Matteo

**SUMMARY OF CONTENTS**

**Table S1.** “Strengthening the Reporting of Observational Studies in Epidemiology (STROBE)” statement guidelines [page 3-5]

**Figure ESM1.** Intracranial pressure (ICP), respiratory system compliance (Crs), Plateau pressure (Pplat), and partial pressure of oxygen/inspired fraction of oxygen ratio (PaO2/FiO2) at PEEP of 5 and 15 cmH_2_O [page 6]

**Figure ESM2.** Scatterplots showing the linear association and correlation between Δ respiratory system compliance (Crs), Δmean arterial pressure (MAP) Δcarbon dioxide (PaCO_2_) (lower panel), vs Δ intracranial pressure (ICP) at different study timepoints [page 7]

**Figure ESM3.** Scatterplots showing the linear association and correlation between ΔPplat vs Δ intracranial pressure (ICP) at different study timepoints. Dotted lines represent the 95% confidence intervals for the linear regression [page 8]

**Table S1.** “Strengthening the Reporting of Observational Studies in Epidemiology (STROBE)” statement guidelines.

|  | **Item No.** | **Recommendation** | **Page  No.** | **Relevant text from manuscript** |
| --- | --- | --- | --- | --- |
| **Title and abstract** | 1 | (*a*) Indicate the study’s design with a commonly used term in the title or the abstract | 1-2 |  |
|  |  | (*b*) Provide in the abstract an informative and balanced summary of what was done and what was found | 2 |  |
| **Introduction** | | | |  |
| Background/rationale | 2 | Explain the scientific background and rationale for the investigation being reported | 4 |  |
| Objectives | 3 | State specific objectives, including any prespecified hypotheses | 5 |  |
| **Methods** | | | |  |
| Study design | 4 | Present key elements of study design early in the paper | 5-6 |  |
| Setting | 5 | Describe the setting, locations, and relevant dates, including periods of recruitment, exposure, follow-up, and data collection | 5-6 |  |
| Participants | 6 | (*a*) *Cohort study*—Give the eligibility criteria, and the sources and methods of selection of participants. Describe methods of follow-up | 5-6 |  |
|  |  |  |  |  |
| Variables | 7 | Clearly define all outcomes, exposures, predictors, potential confounders, and effect modifiers. Give diagnostic criteria, if applicable | 6-9 |  |
| Data sources/ measurement | 8* | For each variable of interest, give sources of data and details of methods of assessment (measurement). Describe comparability of assessment methods if there is more than one group | *6-9* |  |
| Bias | 9 | Describe any efforts to address potential sources of bias | 5-9 |  |
| Study size | 10 | Explain how the study size was arrived at | 9-10 |  |

| Quantitative variables | 11 | Explain how quantitative variables were handled in the analyses. If applicable, describe which groupings were chosen and why | 9-10 |  |
| --- | --- | --- | --- | --- |
| Statistical methods | 12 | (*a*) Describe all statistical methods, including those used to control for confounding | 9-10 |  |
|  |  | (*b*) Describe any methods used to examine subgroups and interactions |  |  |
|  |  | (*c*) Explain how missing data were addressed |  |  |
|  |  |  |  |  |
|  |  | (d) Describe any sensitivity analyses |  |  |
| **Results** | | | | |
| Participants | 13* | (a) Report numbers of individuals at each stage of study—eg numbers potentially eligible, examined for eligibility, confirmed eligible, included in the study, completing follow-up, and analysed | 10-11 |  |
|  |  | (b) Give reasons for non-participation at each stage |  |  |
|  |  | (c) Consider use of a flow diagram |  |  |
| Descriptive data | 14* | (a) Give characteristics of study participants (eg demographic, clinical, social) and information on exposures and potential confounders | 10-12 |  |
|  |  | (b) Indicate number of participants with missing data for each variable of interest |  |  |
|  |  | (c) *Cohort study*—Summarise follow-up time (eg, average and total amount) |  |  |
| Outcome data | 15* | *Cohort study*—Report numbers of outcome events or summary measures over time | 10-12 |  |
|  |  | *Case-control study—*Report numbers in each exposure category, or summary measures of exposure |  |  |
|  |  | *Cross-sectional study—*Report numbers of outcome events or summary measures |  |  |
| Main results | 16 | (*a*) Give unadjusted estimates and, if applicable, confounder-adjusted estimates and their precision (eg, 95% confidence interval). Make clear which confounders were adjusted for and why they were included | 10-12 |  |
|  |  | (*b*) Report category boundaries when continuous variables were categorized |  |  |
|  |  | (*c*) If relevant, consider translating estimates of relative risk into absolute risk for a meaningful time period |  |  |
| Other analyses | 17 | Report other analyses done—eg analyses of subgroups and interactions, and sensitivity analyses | ESM |  |
| **Discussion** |  |  |  |  |
| Key results | 18 | Summarise key results with reference to study objectives | 12 |  |
| Limitations | 19 | Discuss limitations of the study, taking into account sources of potential bias or imprecision. Discuss both direction and magnitude of any potential bias | 12-16 |  |
| Interpretation | 20 | Give a cautious overall interpretation of results considering objectives, limitations, multiplicity of analyses, results from similar studies, and other relevant evidence | 12-16 |  |
| Generalisability | 21 | Discuss the generalisability (external validity) of the study results | 12-16 |  |
| **Other information** |  |  |  |  |
| Funding | 22 | Give the source of funding and the role of the funders for the present study and, if applicable, for the original study on which the present article is based. | 18 |  |

*Give information separately for cases and controls in case-control studies and, if applicable, for exposed and unexposed groups in cohort and cross-sectional studies.

**Note:** An Explanation and Elaboration article discusses each checklist item and gives methodological background and published examples of transparent reporting. The STROBE checklist is best used in conjunction with this article (freely available on the Web sites of PLoS Medicine at http://www.plosmedicine.org/, Annals of Internal Medicine at http://www.annals.org/, and Epidemiology at http://www.epidem.com/). Information on the STROBE Initiative is available at www.strobe-statement.org

**Figure ESM 1.** Intracranial pressure (ICP), respiratory system compliance (Crs), Plateau pressure (Pplat), and partial pressure of oxygen/inspired fraction of oxygen ratio (PaO2/FiO2) at PEEP of 5 and 15 cmH_2_O.

Black dots and lines represent individual patient data. PEEP: positive end-expiratory pressure.


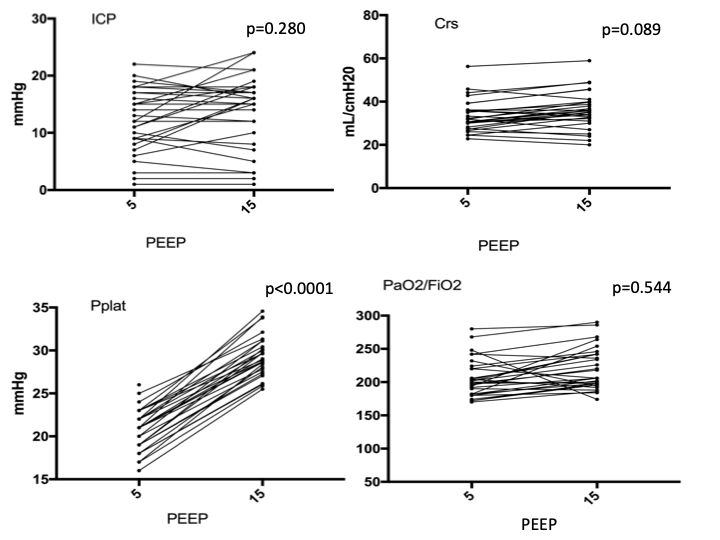


**Figure ESM2.** Scatterplots showing the linear association and correlation between Δ respiratory system compliance (Crs) (left upper panel), Δmean arterial pressure (MAP), mmHg (right upper panel), Δ carbon dioxide (PaCO_2_), mmHg(lower panel)vs Δ intracranial pressure (ICP)mmHg at different study timepoints. Dotted lines represent the 95% confidence intervals for the linear regression.


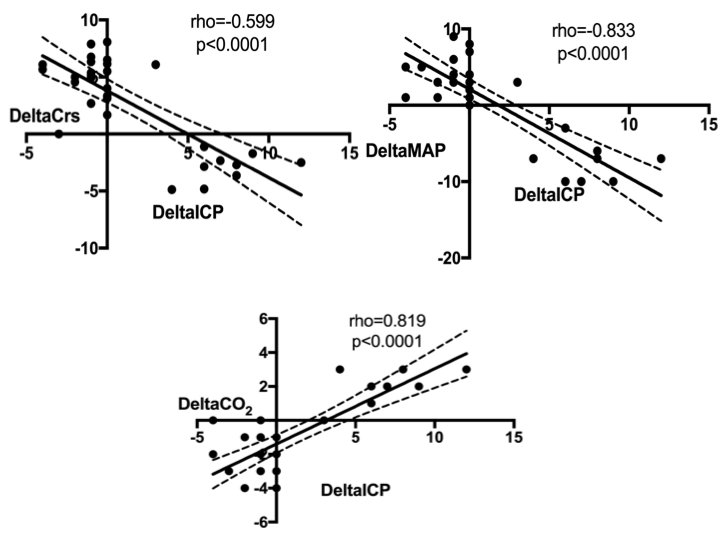


**Figure ESM3.** Scatterplot showing the linear association and correlation between ΔPplat vs Δ intracranial pressure (ICP) at different study timepoints. Dotted lines represent the 95% confidence intervals for the linear regression.

**
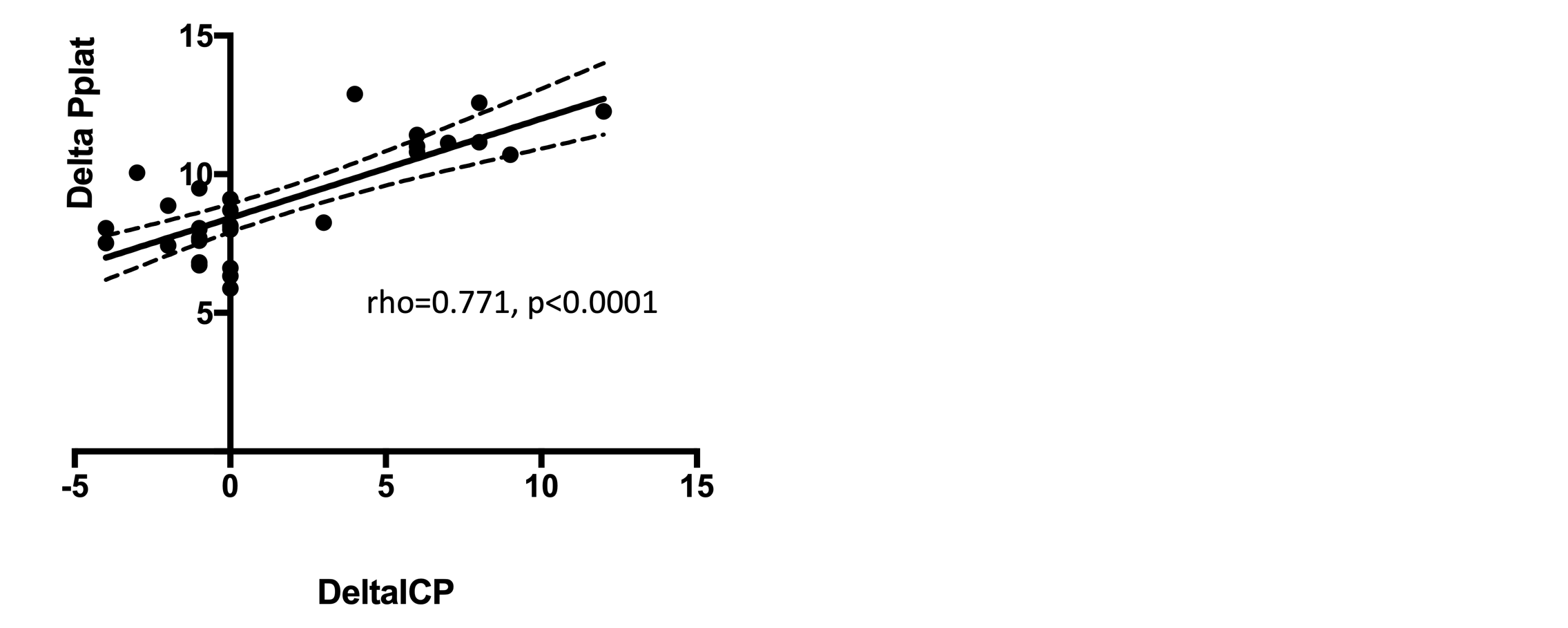
**
